# Evidence for the Use of Mucus Swabs to Detect *Renibacterium salmoninarum* in Brook Trout

**DOI:** 10.3390/pathogens10040460

**Published:** 2021-04-12

**Authors:** Tawni B. Riepe, Victoria Vincent, Vicki Milano, Eric R. Fetherman, Dana L. Winkelman

**Affiliations:** 1 Colorado Cooperative Fish and Wildlife Research Unit, Colorado State University, 1484 Campus Delivery, Fort Collins, CO 80523, USA; 2 Colorado Parks and Wildlife, Aquatic Animal Health Laboratory, 122 East Edison Street, Brush, CO 80723, USA; Victoria.Vincent@state.co.us (V.V.); Vkarstoft@gmail.com (V.M.); 3 Colorado Parks and Wildlife, Aquatic Wildlife Research Section, 317 West Prospect Road, Fort Collins, CO 80525, USA; Eric.Fetherman@state.co.us; 4 U.S. Geological Survey, Colorado Cooperative Fish and Wildlife Research Unit, 1484 Campus Delivery, Fort Collins, CO 80523, USA; Dana.Winkelman@colostate.edu

**Keywords:** non-lethal, brook trout, *Renibacterium salmoninarum*, bacterial kidney disease, aquatic pathogens

## Abstract

Efforts to advance fish health diagnostics have been highlighted in many studies to improve the detection of pathogens in aquaculture facilities and wild fish populations. Typically, the detection of a pathogen has required sacrificing fish; however, many hatcheries have valuable and sometimes irreplaceable broodstocks, and lethal sampling is undesirable. Therefore, the development of non-lethal detection methods is a high priority. The goal of our study was to compare non-lethal sampling methods with standardized lethal kidney tissue sampling that is used to detect *Renibacterium salmoninarum* infections in salmonids. We collected anal, buccal, and mucus swabs (non-lethal qPCR) and kidney tissue samples (lethal DFAT) from 72 adult brook trout (*Salvelinus fontinalis*) reared at the Colorado Parks and Wildlife Pitkin Brood Unit and tested each sample to assess *R. salmoninarum* infections. Standard kidney tissue detected *R. salmoninarum* 1.59 times more often than mucus swabs, compared to 10.43 and 13.16 times more often than buccal or anal swabs, respectively, indicating mucus swabs were the most effective and may be a useful non-lethal method. Our study highlights the potential of non-lethal mucus swabs to sample for *R. salmoninarum* and suggests future studies are needed to refine this technique for use in aquaculture facilities and wild populations of inland salmonids.

## 1. Introduction

Disease outbreaks disrupt fish production efforts by reducing the number of fish cultured, the number available for stocking into the wild or delivered for consumption, and the ability to move fish among hatcheries or from the hatchery to wild populations. To reduce disease outbreaks at aquaculture facilities, it is crucial to monitor fish health and to detect the presence of regulated, virulent pathogens. Many of the current American Fisheries Society Fish Health Blue Book (AFS-FHS; [1]) protocols to detect pathogens require lethal sampling. However, lethal sampling may be undesirable with valuable or rare broodstocks and developing non-lethal diagnostic techniques that allow for consistent detection of pathogens is a high priority. Detection of fish pathogens often entails euthanizing a proportion of the target population to collect organ tissues from an adequate number of hosts and determine the pathogen prevalence within the population. Dependent upon the population or lot size in a rearing facility, a large proportion of fish may need to be tested to estimate the pathogen’s prevalence with a high degree of confidence [2,3].

The development of non-lethal techniques may reduce the need to euthanize fish and be especially valuable for assessing the presence or absence of a pathogen among populations of sensitive or valuable species that cannot be lethally sampled [4]. Non-lethal methods may also allow the testing of more individuals than would be possible with lethal methods, thereby increasing the likelihood that a pathogen is detected. Infection dynamics can also be studied using non-lethal methods through repeated testing over time [5]. For instance, diagnosis of infectious hematopoietic necrosis virus (IHNV) and viral hemorrhagic septicemia virus (VHSV) in rainbow trout (*Oncorhynchus mykiss*) previously required euthanizing fish, but researchers demonstrated the ability to diagnose and track infection status over multiple testing periods using fin clips from the same host [5,6]. Furthermore, surveillance of *Aeromonas salmonicida* in hatchery stocks of Atlantic salmon (*Salmo salar*) utilized non-lethal mucus swabs for early detection of *A. salmonicida* leading to proper treatment prior to stocking [7]. The development of more non-lethal detection methods for regulated fish pathogens may substantially benefit surveillance and management in cultured and wild fish populations.

*Renibacterium salmoninarum*, the bacterial pathogen that causes bacterial kidney disease, is a concern in salmonid populations. The bacteria can cause significant pathological effects among infected fishes. However, more often it exists sub-clinically and presents no symptoms of disease, making it difficult to observe signs of illness [8]. Bacterial kidney disease may also cause high mortalities among salmonids at all life stages, albeit with varying susceptibility and overall *R. salmoninarum* prevalence among species [9]. Inland salmonid populations appear to exhibit a higher resistance to disease caused by *R. salmoninarum* infections than many species in the Pacific Northwest, including chinook salmon (*Oncorhynchus tshawytscha*) and coho salmon (*Oncorhynchus kisutch*) [10,11,12], and as such, many *R. salmoninarum* studies have been focused on anadromous salmonid populations. Consequently, non-lethal sampling methods to detect *R. salmoninarum* have not been implemented for use in inland salmonids.

In this study, we evaluated brook trout (*Salvelinus fontinalis*) collected from the Colorado Parks and Wildlife (CPW) Pitkin Brood Unit (Pitkin, Colorado, USA) to address two primary objectives: (1) determine if non-lethal and standard lethal sampling methods give similar predictions of *R. salmoninarum* presence, and (2) determine which non-lethal sampling method has the highest rate of predicting infection status when the infection status is known using standard lethal diagnostic techniques. Specifically, we collected and evaluated kidney tissue and compared those results to non-lethal anal, buccal, and mucus swabs from 72 adult brook trout.

## 2. Results

### 2.1. Assay Performance

Among the 72 brook trout collected from the CPW Pitkin Brood Unit, 21 were positive with single-round PCR (PCR), 47 were positive with direct fluorescent antibody test (DFAT), and 50 were positive using quantitative PCR (qPCR). The overall estimated detection probabilities for PCR, DFAT, and qPCR were 0.24, 0.70, and 0.74, respectively. Pairwise contrasts indicated that DFAT predicted the presence of *Renibacterium salmoninarum* 7.27 times more often than PCR (*p* < 0.01), and qPCR 9.26 times more often than PCR (*p* < 0.01). No significant difference in detecting *R. salmoninarum* was observed between DFAT and qPCR (*p* = 0.82; Table 1). Given the low detection probability for PCR, and similarity in the diagnostic capabilities of DFAT and qPCR, PCR was dropped from further analyses. As such, subsequent subsections highlight results for the tissues tested rather than assay type used.

### 2.2. Tissue Comparisons

**Figure 1:**
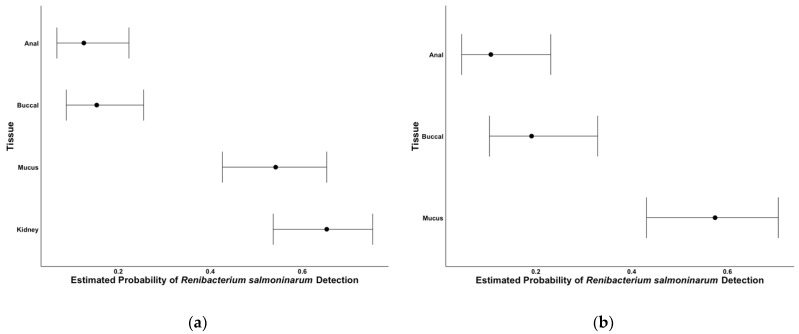
Model-based detection probability estimates (95% confidence interval bars) for *Renibacterium salmoninarum*; (**a**) using anal, buccal and mucus swabs, or kidney tissues, and (**b**) using anal, buccal, or mucus swabs when samples are known to be positive by testing kidney tissues with DFAT.

Among the 72-brook trout collected, 47 fish (65.3%) were determined positive for *R. salmoninarum* using DFAT on kidney tissue samples. Positive detections by qPCR of nonlethal swab sampling with anal, buccal, and mucus swabs were 9 (11.1%), 11 (15.3%), and 39 (54.2%), respectively. *Renibacterium salmoninarum* infections among kidney tissue had the highest detection probability (Figure 1a). Among the non-lethal tissues, we were more likely to detect the bacteria using mucus swabs. *Renibacterium salmoninarum* detection probabilities also suggest kidney tissues and mucus swabs are the best tissues to sample, especially compared to anal and buccal swabs (Figure 1a). Furthermore, kidney tissue and mucus swab detection results had low odds of differing from one another (Table 1), indicating that mucus swabs are at least as effective as kidney samples in detecting *R. salmoninarum*. Buccal or anal swabs had much higher odds of differing from kidney tissue detection of *R. salmoninarum*, suggesting they may not be an appropriate non-lethal method for detection of the bacteria (Table 1).

### 2.3. Comparisons When Kidney Tissue Is Positive

Forty-seven fish were determined positive using DFAT analysis of kidney tissue. Of those 47 fish, 5 anal (10.6%), 9 buccal (19.2%), and 28 mucus (57.5%) samples were considered positive for *R. salmoninarum* using qPCR. Mucus swabs were the best non-lethal sampling method for determining if a fish was positive for *R. salmoninarum* when kidney tissue was positive compared to anal or buccal swabs (11.34 or 5.70 higher odds, respectively; Table 1). Likewise, the probabilities of detecting *R. salmoninarum* indicated that mucus swabs were more likely to detect the bacteria than anal or buccal swabs (Figure 1b). Interestingly, when the bacterium was not detected in kidney tissues by DFAT, mucus swabs detected *R. salmoninarum* in an additional 11 fish, anal swabs in an additional 2 fish, and buccal swabs in 1 additional fish.

## 3. Discussion

The utilization of non-lethal methods is not well-developed for determining the presence or absence of *Renibacterium salmoninarum* in cultured or wild salmonid populations and, particularly, in inland salmonids. Therefore, evaluating the performance of non-lethal sampling methods for detecting and predicting the presence of *R. salmoninarum* is essential for validating and advancing their use in inland salmonid populations. Overall, our results indicate using kidney tissues (DFAT) and mucus swabs (qPCR) to test for *R. salmoninarum* in brook trout offers the highest detection probabilities for the tissues tested in this study and are equally effective. Mucus swabs were also the best non-lethal sampling method for detecting *R. salmoninarum* when the fish was positive for the bacteria by testing kidney tissues (DFAT).

Confidently detecting pathogens often requires sacrificing a large number of fish [2,3], but this is undesirable, particularly with species of high conservation concern. Ovarian fluid from spawning adult female fish has been used to detect pathogens, such as *R. salmoninarum*, non-lethally; however, this is limited to fish that are mature, gravid, and female [13]. Most fish are not typically held until they reach spawning maturity, limiting the usefulness of testing ovarian fluid. In previous studies, chinook salmon as young as 6 months old have been known to be naturally and/or experimentally infected [14,15,16], and inland rainbow trout have tested positive for *R. salmoninarum* as soon as 11 days after swim-up [17]. Therefore, non-lethal sampling methods are needed for all age classes and maturity statuses. Our study suggests using mucus swabs as a sampling method, coupled with a qPCR assay, could be useful as a screening tool for *R. salmoninarum*. Our data also suggest that mucus swabs coupled with qPCR are at least as effective as the standard kidney test, although additional controlled research with more species and testing at various time points of infection and infection level is needed.

Interestingly, mucus swabs detected *R. salmoninarum* in 11 fish that were negative by kidney tissue testing. It is possible that some of the positive mucus detections in our study could reflect bacteria present in the water and represent an exposure or subclinical infection but not an internal infection [18,19]. Mucus is a primary defensive mechanism of fish and can be shed and replaced to prevent the colonization of bacterial pathogens and active infections [20]. We did not observe any external signs of disease and were therefore unable to include an ordinal visual disease assessment in this study and relate signs of disease to a positive mucus swab. This is typical because *R. salmoninarum* causes a systemic, slow-progressing disease with varying symptoms, which reduces the probability of visually observing signs of acute and sub-acute symptoms [1,21,22]. Therefore, further experiments are needed to understand the meaning of a positive mucus swab. Positive results may indicate (1) a future infection, following attachment to the underlying dermis tissues in the mucus layer; (2) a previous or active infection, and the mucus may be aiding in clearing the bacteria from the fish; or, (3) the bacteria are present in the fish’s environment, but detection is not indicative of a previous or future infection. Despite uncertainty about the status of fish testing positive with mucus swabs, they may advantageously be used to determine if *R. salmoninarum* is present in the environment. In the case of anadromous or other migrating salmonids [23], mucus swabs could present a means for determining whether or not fish traveled through areas where *R. salmoninarum* was present. Additionally, mucus swabs could allow the initial screening of wild fish that are being collected to supplement hatchery broodstocks for conservation purposes, and those fish could remain in isolation to prevent active transmission of *R. salmoninarum* into a hatchery unit.

While mucus swabs show promise for detecting *R. salmoninarum*, they may not be well-suited for testing all fish species or during certain life history periods. For example, the skin of anadromous salmonids and brown trout (*Salmo trutta*) is known to thicken during their spawning migration, which reduces the amount of mucus secreted [24,25]. When mucus is not being replaced during spawning, mucus may not be indicative of the internal infection status of the fish. Anal and buccal swabs may also be affected by the timing of sampling and this may be why we determined that anal and buccal swabs were not effective ways to sample for *R. salmoninarum*. For example, buccal swab effectiveness may be dependent on fish feeding and ingesting bacteria. Therefore, if fish are not feeding, then results from buccal swabs would be negative despite potential exposure. Fish ingesting *R. salmoninarum* could potentially lead to an infection through horizontal transmission [18,26,27]. Similarly, timing issues are possible with anal swab effectiveness and may have led to our inference that they are not effective. Positive anal swabs may be dependent on the fish actively shedding the bacteria [14], and a negative test may be misleading relative to the internal infection status of the fish. Clearly, more studies are needed to address potential issues regarding the timing of non-lethal sampling versus the level of infection, but we feel that mucus testing offers the most promising avenue for non-lethal testing among inland salmonids.

Our study suggests that single-round PCR, using the specified primers [17], has a lower probability of detecting *R. salmoninarum* than standard lethal methods (DFAT and qPCR) used by the AFS-FHS [1]. Our conclusions are limited by our opportunistic study design that conducted assays at different time points, and a lack of additional tissue prevented rerunning samples with qPCR. The discrepancy between single-round PCR and qPCR may be related to the sensitivity and specificity of each assay [17,18,28,29]. For instance, nested PCR shows lower diagnostic sensitivity and specificity probabilities from qPCR when testing kidney tissues for *R. salmoninarum* [29]. However, the sensitivity and specificity for single-round PCR is not well known for *R. salmoninarum* in kidney tissues, as there has only been one study using this method [17]. Future studies would benefit from using the same assays on the same tissues. This would allow us to optimize assay conditions and understand the reliability of these assays to detect *R. salmoninarum*, especially when comparing non-lethal to lethal sampling methods. We also recognize that due to our study design, our sample size is relatively small, and our analyses could be influenced by the low number of known positive samples.

Our study offers a first step in utilizing non-lethal methods to detect *R. salmoninarum* in inland trout. Non-lethal sampling methods could be valuable in determining the presence of *R. salmoninarum* in populations of rare and vulnerable species and in aquaculture facilities where there may not be enough fish available for lethal testing. Therefore, the addition of non-lethal sampling techniques could allow for multiple testing, the monitoring of infections, and, potentially, the fate of infections within populations, allowing for a more nuanced understanding of the consequences of *R. salmoninarum* infections.

## 4. Materials and Methods

### 4.1. Fish and Tissue Collection

We sampled 72 brook trout from the CPW Pitkin Brood Unit (UTM coordinates: Easting 366588, Northing 4272824, Zone 13S) on 3 May 2017. Average spring water temperature where the fish were located on the unit was 5.3 °C. Fish were intentionally selected from a lot of adult brook trout with an ongoing *Renibacterium salmoninarum* infection. Size and sex of fish were not recorded as this was an opportunistic collection. Three non-lethal samples and one lethal sample were collected from each fish. Non-lethal samples included individual swabs of anal and buccal areas as well as swabbing of the lateral line on both sides of the fish for mucus collection. Swabs were collected by firmly running a 2 mm-diameter, sterile, cotton-tipped applicator along each of the three surfaces ten times, depositing each swab into individual, sterile, 4-mL collection tubes, and placing them on dry ice. Following swab collection, fish were euthanized through immersion in tricaine methanesulfonate (MS-222; Syndel) for 10 to 15 min. Lethal samples consisted of whole kidney tissue collected through an abdominal incision and placed into sterile Whirl-Pak bags on dry ice for transport. All samples were maintained at −20 °C until processed.

### 4.2. Laboratory Analyses

Kidney tissue samples were prepared for analyses as part of a routine fish health inspection via direct fluorescent antibody test (DFAT) and single-round PCR; qPCR is not used during Colorado state health inspections. In addition, current standard techniques [1] declare DFAT of kidney tissue can only be accomplished using lethal testing and not to evaluate non-lethal swabs. Extractions of DNA from each kidney sample (approximately 0.25 g) were completed using the Qiagen DNeasy Blood and Tissue Kit protocol (Hilden, Germany) with known positive and negative control tissue samples. Single-round PCR was used to determine the presence of *R. salmoninarum* DNA in kidney tissues, with Forward 5′-TTTGGGGTGGCTCCTCTTGCG-3′, PM14, and Reverse 5′-ATTGGGGATGGCGCATTATCG-3′, PM15 primers targeting the major soluble antigen gene (*msa*; P57 protein) for amplification, and visual confirmation of band formation of the 377 base pair product [17]. Kidney tissues were prepared for DFAT by making tissue imprints from each fish on a 12-well slide. Slides were stained utilizing a *Fluorescein*-labeled, affinity purified polyclonal antibody to *R. salmoninarum* (KPL; Milford, MA, USA) with eriochrome black T counterstain [30]. Slides were examined at 500 times magnification with a fluorescein isothiocyanate (FITC) fluorescent lamp at a wavelength of 400 nm. Tissue imprints showing visible fluorescent cells were further examined at 1000 times magnification to confirm identification through cell morphology and size [1].

Anal, buccal, and mucus swabs were prepared for analysis via high throughput, real-time qPCR. DNA extraction was similarly completed using Qiagen DNeasy Blood and Tissue Kit protocols. Additionally, we followed the Qiagen protocol for Gram-positive bacterial swabs, and the DNA elution step was increased with 200 μL of AE buffer [29]. We established standard curves for quantification by creating ten-fold serial dilutions of *R. salmoninarum* from pure bacterial culture grown in KDM2 broth at 15 °C for 9 days. Our positive controls ranged from 1.1 × 10^5^ to 1.1 × 10 bacterial cells. The qPCR cut-off Cq value was determined to be 37.75 (Riepe unpublished data), which is considered an acceptable value [31]. Quantitative PCR was performed using ABI StepOnePlus System (Applied Biosystems, Foster City, CA, USA) to detect the *msa* gene in a final volume of 25 μL of DNA template and 12.5 μL of TaqMan Gene Expression Master Mix with primer sets RS 1238 Forward, 5′-GTGACCAACACCCAGATATCCA-3′, and RS 1307 Reverse, 5′-TCGCCAGACCACCATTTACC-3′, and MGB probe 1262, 5′-CACCAGATGGAGCAAC-3′ [32].

### 4.3. Data Analyses

We used a generalized linear mixed model (GLMM) to analyze pathogen detection [33]. The presence of *R. salmoninarum* was treated as a continuous, binomially distributed response variable in a GLMM with a logit link, using the glmer function in the lme4 package in R to perform multiple pairwise comparisons [34].

Detections of *R. salmoninarum* can differ among assays [31], including the assays used in this study (PCR, qPCR, and DFAT). We first compared the ability of each assay to predict the presence of *R. salmoninarum* in our samples. We characterized a positive assay result as a binomial response and each diagnostic assay type as a predictor (PCR, qPCR, and DFAT). Individual fish were included as a random intercept term to account for the repeated, non-independent observations on each fish (three assay types per fish: n = 216 observations from 72 brook trout). We report these findings as probabilities of detection, odds ratio contrasts obtained from the emmeans package, standard error (SE), and *z*- and *p*-values (α = 0.05) with a Tukey adjustment for small sample size.

We compared the utility of each tissue in predicting the presence of *R. salmoninarum*. As predictor variables, we included kidney tissue and anal, buccal, and mucus swabs, with individual fish included as a random effect. All four tissues were tested from each fish, resulting in 288 observations from 72 brook trout. We report the probability of detection, odds ratios, SE, and *z*- and *p*-values (α = 0.05) for each tissue.

A GLMM was also used to evaluate which non-lethal sampling method(s) best predicted an infection when the kidney tissue was positive by DFAT. The presence of *R. salmoninarum* was the response variable, as determined by positive kidney tissues, and anal, buccal, and mucus swabs were included as predictors. Individual fish were a random intercept term to account for the repeated and non-independent observations on each fish (three tissues tested per fish; n = 141 observations from 47 *R. salmoninarum*-positive brook trout). Similar to the analyses above, odds ratios and estimated detection probabilities are reported and used to assess the capacity of a non-lethal tissue to predict a known positive infection status.

## Figures and Tables

**Table 1:** Results are based on three separate logistic regression models in which the presence of *Renibacterium salmoninarum* was treated as a binomial response variable. Individual fish were treated as random intercept terms. Model 1 (Assay Performance) accounts for 216 observations from 72 brook trout, model 2 (Tissue Comparisons) accounts for 288 observations from 72 brook trout, and model 3 (Comparisons when Kidney Tissue is Positive) accounts for 141 observations from 47 brook trout. Pairwise contrasts are given for each of the models, including odds ratios, standard error (SE), and *z*- and *p*-values (α = 0.05) for each contrast.

Model	Contrasts	Odds Ratio	SE	*z*-Value	*p*-Value
Assay Performance	DFAT/PCR	7.27	3.35	4.30	<0.01
	DFAT/qPCR	0.78	0.32	−0.60	0.82
	qPCR/PCR	9.26	4.43	4.65	<0.01
Tissue Comparisons	Kidney/Anal	13.16	5.71	5.94	<0.01
	Kidney/Buccal	10.43	4.28	5.71	<0.01
	Kidney/Mucus	1.59	0.54	1.36	0.53
	Mucus/Anal	8.27	3.54	4.94	<0.01
	Mucus/Buccal	6.55	2.65	4.65	<0.01
Comparisons when Kidney Tissue is Positive	Mucus/Anal	11.34	6.32	4.36	<0.01
	Mucus/Buccal	5.70	2.70	3.67	<0.01
	Buccal/Anal	1.99	1.20	1.14	0.49

## Data Availability

The data available in this study are available on request from the corresponding author. The data are not publicly available due to ethical reasons.
